# Induction of Viable but Non-Culturable State in Clinically Relevant Staphylococci and Their Detection with Bacteriophage K

**DOI:** 10.3390/antibiotics12020311

**Published:** 2023-02-02

**Authors:** Katja Šuster, Andrej Cör

**Affiliations:** 1 Department of Research, Valdoltra Orthopaedic Hospital, 6280 Ankaran, Slovenia; 2 Faculty of Education, University of Primorska, 6000 Koper, Slovenia

**Keywords:** phage typing, molecular detection, bacteriophages, VBNC, pathogenic bacteria, PJI, biofilm

## Abstract

Prosthetic joint infections are frequently associated with biofilm formation and the presence of viable but non-culturable (VBNC) bacteria. Conventional sample culturing remains the gold standard for microbiological diagnosis. However, VBNC bacteria lack the ability to grow on routine culture medium, leading to culture-negative results. Bacteriophages are viruses that specifically recognize and infect bacteria. In this study, we wanted to determine if bacteriophages could be used to detect VBNC bacteria. Four staphylococcal strains were cultured for biofilm formation and transferred to low-nutrient media with different gentamycin concentrations for VBNC state induction. VBNC bacteria were confirmed with the BacLight^TM^ viability kit staining. Suspensions of live, dead, and VBNC bacteria were incubated with bacteriophage K and assessed in a qPCR for their detection. The VBNC state was successfully induced 8 to 19 days after incubation under stressful conditions. In total, 6.1 to 23.9% of bacteria were confirmed alive while not growing on conventional culturing media. During the qPCR assay, live bacterial suspensions showed a substantial increase in phage DNA. No detection was observed in dead bacteria or phage non-susceptible *E. coli* suspensions. However, a reduction in phage DNA in VBNC bacterial suspensions was observed, which confirmed the detection was successful based on the adsorption of phages.

## 1. Introduction

In the last decade, research on bacteriophage use has increased for diagnostic and therapeutic purposes in human medicine [1]. Bacteriophages are viruses that specifically recognize and attach to their target host bacteria, inject their genetic material, reproduce inside the host, and release their progeny by lysing the bacterium. Due to the extraordinary specificity of phages for a particular genus, species, or even strain of bacteria, they could be a promising new tool for the rapid detection of bacteria in clinical samples. Bacteriophage K is a broad-spectrum staphylococcal bacteriophage [2,3,4,5]. Previous research work showed that combining bacteriophage K with qPCR technology enabled the detection of staphylococci in the sonicate fluid of infected prosthetic joints [6,7].

The replacement of joints with artificial joint prostheses is one of the most successful surgical procedures in orthopedic surgery as it has improved the life quality of millions of patients by saving them from chronic pain and improving their mobility. Due to technological advances, the number of prostheses that need to be replaced is decreasing; however, it is still necessary to replace approximately 10% of them [8]. The most serious complication leading to joint prosthesis revision is bacterial prosthetic joint infection (PJI), which is reported to occur in 0.8 to 1.9% of knee arthroplasties and 0.3 to 1.7% of hip arthroplasties [9]. The microbiological culturing of samples represents the gold standard for establishing the microbiological diagnosis (microbial typing) of PJI but does not always identify the pathogen. Culture-negative infections have been widely reported in the literature and account for about 7–42% [10,11,12,13] of PJI cases, occurring at a higher rate in late PJI. Culture-negative infections unable the selection of targeted antimicrobials; instead, broad-spectrum or multiple antibiotics are administered to cover the most common microorganisms, which can be less effective and lead to treatment failure. Failure to isolate the causative pathogen by culture is mostly due to antibiotic administration prior to sampling, infections with fastidious or uncommon microorganisms, improper sampling and lack of resuscitation in the laboratory, and biofilm formation on biomaterial. Biofilms are associated with the persistence and chronicity of infections and represent an important threat to human health, especially due to their increased virulence and tolerance to antimicrobials.

The inaccurate use of antibiotics due to misdiagnosis or the administration of inadequate doses importantly contributes to the emerging multidrug-resistant strains, and may induce persistence and a VBNC state in bacteria [14,15]. As for antibiotic pressure, other environmental conditions can also act as stressors on bacterial cells. Within layers of a biofilm, different stressful microenvironments form that are low in oxygen and nutrients and have a low pH due to higher waste concentrations. Such conditions promote phenotypic heterogeneity within bacterial populations in a biofilm, giving rise to persisters and VBNC bacteria [14,16,17]. As a response to stressors, bacteria activate a cascade of stress-related mechanisms followed by important morphological changes and lower metabolic activity. The transition of bacteria into those states is generally accepted as a bacterial mechanism of survival.

Due to all the changes that bacteria undergo while transitioning to the VBNC state, they also lose their ability to grow on routinely used bacteriological media [18,19,20]. Currently, there is no validated and specific method for the identification of all viable bacteria in clinical specimens. This leads to false negative results due to a lack of growth by conventional microbiological culturing methods or underestimation of the total bacterial number or species in a sample. Under continued stressful conditions, bacteria can remain in the VBNC state over long periods. However, during the process known as resuscitation, bacteria can revive into the culturable state, regaining full metabolic activity and virulence, and thus causing a recurrence of infection in patients weeks or months later [21]. Therefore, the underestimation or non-detection of bacteria due to biofilm formation on medical devices and the presence of VBNC bacteria pose a great risk to public health.

Identifying the causative pathogen is of the utmost importance for an optimal outcome in treating implant-related infections as it directly influences the choice of antimicrobial and surgical therapy. However, further studies for the detection of all viable bacteria in clinical samples are needed. In this study, we demonstrate that phage K combined with qPCR enables the detection of VBNC staphylococci within biofilms. The use of bacteriophages for bacterial typing could therefore represent an important alternative to conventional microbiological diagnostic procedures that will allow the specific detection of all viable bacteria in clinical specimens.

## 2. Results

### 2.1. Biofilm Formation and Induction of VBNC State

Minimal inhibitory concentrations (MIC) of gentamycin were determined to be 3.12 µg/mL for *S. aureus* ATCC 25923, *S. epidermidis* DSM 3269, and *S. lugdunensis* OBV 20/143, whereas for the *S. aureus* OBV 17/166 strain, the MIC was determined to be 12.5 µg/mL.

**Figure 1:**
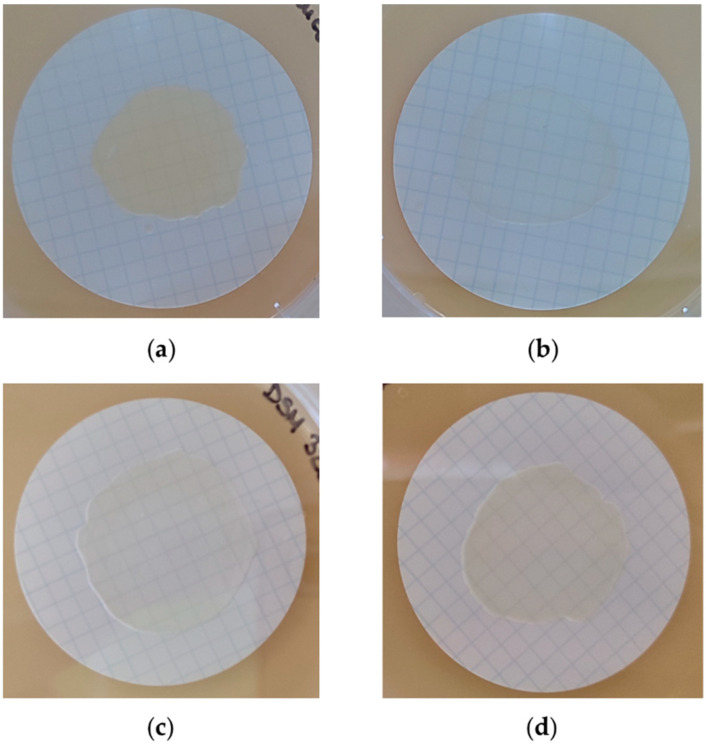
Biofilm formation on ø 47 mm membranes placed on BHI agar plates with 1% glucose: (**a**) *S. aureus* OBV 17/166; (**b**) *S. aureus* ATCC 25923; (**c**) *S. epidermidis* DSM 3269; (**d**) *S. lugdunensis* OBV 20/143.

All strains successfully formed biofilms on filter paper membranes during the 48 h incubation on BHI agar plates supplemented with 1% glucose. The biofilms’ morphology was observed macroscopically and differed between strains. *S. aureus* OBV 17/166 biofilms appeared thicker and retained a more intense yellow color than other bacterial biofilms. *S. aureus* ATCC 25923 formed thinner biofilms than other staphylococcal strains (Figure 1).

The VBNC state in all tested staphylococcal strains was successfully induced in vitro by starvation on a low-nutrient M9 medium and antibiotic pressure using different concentrations of gentamycin. All control biofilms exposed only to starvation without gentamycin still retained their culturability after 30 days of exposure. Results are presented in Table 1.

*S. aureus* ATCC 25923 was the first strain that stopped growing on a nutrient medium regardless of the antibiotic concentration used. The loss of culturability was first observed after 8.0 ± 1.0 days of starvation and exposure to gentamycin at an 8 × MIC. No correlation was observed between the used concentrations and time to growth cessation in both tested *S. aureus* strains. In other tested strains, a correlation between antibiotic concentration and the exposure time until culturability loss has been found. There was a statistically significant difference between the use of 16 × MIC and 8 × MIC or 4 × MIC in *S. epidermidis* (*p* < 0.001) and *S. lugdunensis* (*p* = 0.014 and *p* = 0.003, respectively), but not between the use of 4 × MIC and 8 × MIC (*p* = 0.05), analyzed with the Newman–Keuls method.

**Figure 2:**
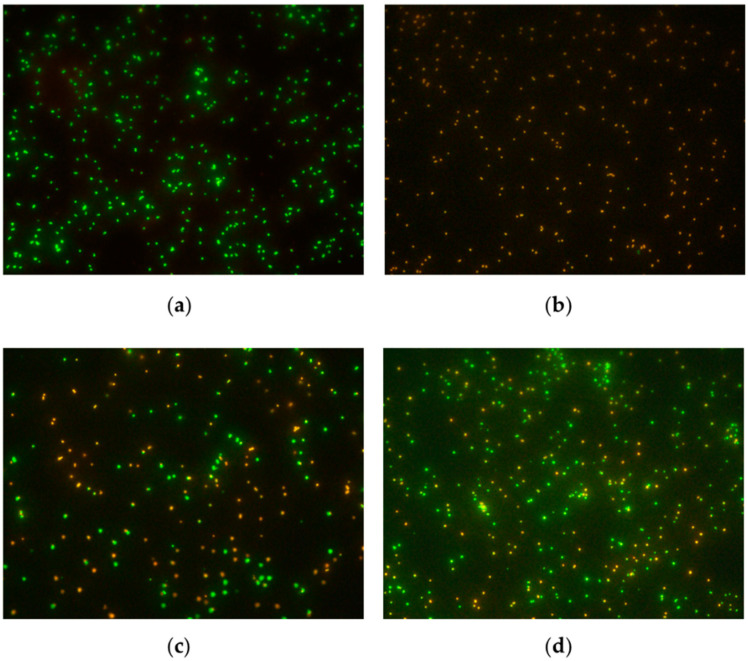
Examples of viable (green) and dead (red) bacteria (*S. epidermidis* DSM 3269) visualized using fluorescence microscopy: (**a**) suspension of live bacteria; (**b**) suspension of dead bacteria; (**c**) suspension of induced VBNC bacteria by gentamycin at 4 × MIC; (**d**) suspension of induced VBNC bacteria by gentamycin at 16 × MIC. Magnification, 600×.

The presence of VBNC bacteria was confirmed after staining bacterial suspensions with the BacLight™ kit followed by visualization with fluorescence microscopy (Figure 2) and spectrophotometric measurements of emitted green and red fluorescence. The percentages of live bacteria present in samples are presented in Table 1.

The percentage of live bacteria in suspensions where the VBNC state was induced by exposure to gentamycin at 4 × MIC ranged between 6.1 ± 0.4% and 15.2 ± 3.5%, between 10.7 ± 1.6% and 23.7 ± 0.5% when biofilms were exposed to 8 × MIC of gentamycin, and between 10.3 ± 0.9% and 23.9 ± 0.5% when exposed to 16×MIC of gentamycin. In *S. aureus* ATCC 25923 and *S. lugdunensis* OBV 20/143, no correlation between the percentage of viable bacteria and used gentamycin concentration has been found (*p* = 0.069 and *p* = 0.059, respectively). In *S. epidermidis* and *S. aureus* OBV 17/166 strains, there was a statistically significant difference in the percentage of viable bacteria in samples that were exposed to gentamycin 4 × MIC and 8 × MIC (*p* = 0.02). In *S. aureus* OBV 17/166, there was also a significant difference in the number of viable bacteria when comparing 8 × MIC and 16 × MIC results (*p* = 0.002), whereas in *S. epidermidis* the difference was significant, when comparing 4 × MIC with 16 × MIC (*p* = 0.017).

### 2.2. Detection of VBNC Bacteria with Bacteriophages and qPCR

To evaluate the possibility of the detection of VBNC staphylococci with our previously developed method for the detection of staphylococci from sonicate fluid samples of explanted prosthetic joints [6], suspensions of four different induced VBNC staphylococcal strains were inoculated with phage K and assayed for phage K DNA detection in the qPCR as described in the methods.

**Figure 3:**
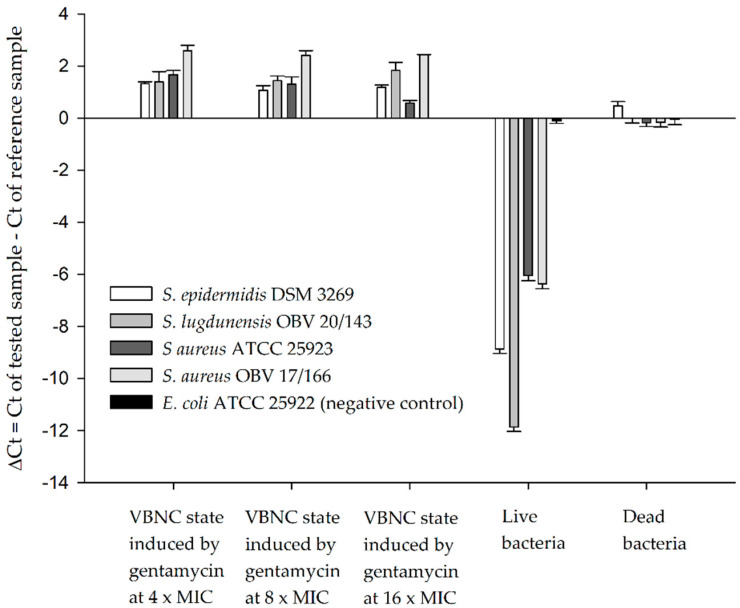
Detection of VBNC, live, and dead bacteria with bacteriophage K and qPCR. Values are expressed as mean ± SD.

During the qPCR assay, a delay in amplification of a specific phage DNA sequence was observed in samples where phage K was added to VBNC bacterial suspensions compared to the reference sample, which contained only the initial amount of added phages. This was due to the phage adsorption on living cells, which did not allow phage multiplication because of their low metabolic state. Suspensions containing only dead bacteria, on the other hand, did not show any statistical difference compared to the reference sample. This indicated that the initial phage DNA was available for qPCR, whether or not the virions adsorbed onto dead cells or cell debris, with a particularity of dead *S. epidermidis*, which showed a statistically significant delay in the amplification of a specific phage sequence. However, the differences between the tested VBNC suspensions and dead bacterial suspensions were statistically different, showing an increased loss of phage DNA in samples where VBNC bacteria were present. For live bacterial suspensions, sequence amplification started 6–12 PCR cycles earlier than the reference control, due to phage proliferation, which was expected since the samples were incubated for 180 min at 37 °C. No difference was observed between the reference sample and live or dead *E. coli* suspensions, which represented the negative control strain, non-susceptible to phage K (Figure 3).

## 3. Discussion

Lytic bacteriophages are virulent bacterial viruses that are gaining interest in modern medicine because of their extraordinary properties, which could be useful both for therapeutic and diagnostic purposes. They can specifically recognize their bacterial host, adsorb to their surface via specific cell receptors, and inject their genetic material into the cell. In that way, they take control of the bacterial metabolism by expressing their genes and assembling new phage particles. During this process, lysins accumulate in the bacterial cell, lysing the bacterium at a certain point, and releasing new progeny phages [22]. For that, more and more studies are focused on possibilities of phage use, not only for treatment but also for the diagnosis of bacterial infections [23]. In this study, we aimed to examine the induction and indirect detection of staphylococci in a low-metabolic state (the VBNC state) with a broad-spectrum staphylococcal bacteriophage (phage K) and qPCR. We demonstrated that phage K combined with qPCR enabled the detection of clinically relevant VBNC staphylococci within biofilms. The detection was based on the adsorption of added phages to VBNC bacteria, which was observed as a delay in phage DNA multiplication in the samples where phage K was added to VBNC bacterial suspensions. The method used in this study was previously developed for the detection of staphylococci in sonicate fluid samples of explanted prosthetic joints [6,7]. Now, we show that both actively growing and VBNC bacteria can be successfully and specifically detected with the bacteriophage-based method in 4 h. The main difference between detecting actively growing bacteria and bacteria in the VBNC state was in the phage adsorption to the bacteria and their reproduction. Bacteriophage K adsorbed and infected all viable bacteria; however, it was able to reproduce only in actively growing bacteria, which resulted in an increase in phage DNA. On the other hand, a decrease in phage DNA was observed in VBNC bacterial suspensions, as phage reproduction strongly depends on the bacterial physiological state [24]. Furthermore, dead bacterial cells were not detected with the method, whether or not the virions could adsorb onto dead cells or cell debris. This was probably due to the sample’s chloroform treatment. Chloroform dissolves the bacterial outer membrane and has been long used for periplasmic protein extraction [25]. The treatment could therefore trigger the release of phages attached to dead bacteria or cell debris, making phage DNA available for qPCR. Chloroform treatment was previously determined to importantly improve the detection of live bacteria with the used method, as it induced the premature lysis of phage-infected bacteria [6]. By disrupting the cell membrane, chloroform provides phage endolysins with access to the peptidoglycan. This triggers the premature lysis of bacteria and the release of bacterial cell content including progeny bacteriophages and/or their components and DNA. However, the results indicate that chloroform did not induce the premature lysis of VBNC bacteria. This is probably because bacteriophages need a live and metabolically active host for their replication, and phage endolysins (or at least enough endolysins) do not form in VBNC bacteria for their lysis. In addition, VBNC bacteria are more resistant to harsh environmental conditions and chemicals, and this could also implicate a higher resistance to chloroform treatment.

Phage selectivity in specific strain recognition minimizes false-negative and false-positive results often associated with detection methods and offers the possibility for the detection of only live bacteria. Phage typing is used to identify bacteria, especially in the food industry, but also in water and environmental pollution. Although the diagnosis with the use of the phage is fast and sensitive, its use in clinical practice is not common [26]. Different already-developed phage-based diagnostic methods are focused on the detection of one bacterial species/strain with a specific bacteriophage. Available FDA-approved and clinically used diagnostic tests are used so far for the identification of *M. tuberculosis*, *Y. pestis*, *B. anthracis,* and *S. aureus* [9].

Culture-negative infections have been widely reported in the literature. Traditional microbiologic culturing does not yield any growth in about 7–42% [10,11,12,13] of PJI cases and occurs at a higher rate in late PJI. In a recent prospective cohort study [27], culture-negative PJI was determined in 19.7% of late PJI and 6.2% of early/delayed PJI cases. The microbiological assessment of device-related biofilm infections improved significantly by sonication fluid culture [28,29,30,31,32]. During sonication, biofilm is detached from the removed device by applying low-intensity ultrasound waves. The culture of sonicate fluid is now widely in use in combination with periprosthetic tissue/synovial fluid cultures to improve the detection of microorganisms in PJI. However, as bacteria in the VBNC state cannot be detected with conventional culturing methods, they contribute importantly to culture-negative results, representing an important clinical issue leading to inadequate antibiotic administration and inadequate patient treatment. For some, the infection goes undetected (silent infections) at first; for others, the prescribed antibiotics (if prescribed) are not needed; and in other cases, patients receive weak epidemiologically inadequate broad-spectrum antibiotics instead of specific ones. Not being able to detect and efficiently treat VBNC bacterial infections contributes to infection recurrence, increases in health care costs, patient dissatisfaction, and importantly to the emerging antimicrobial resistance, which is becoming more and more common today.

The detection of VBNC bacteria remains a challenge in clinical practice, and there is a need for a new specific and sensitive method for the quick detection of non-culturable bacteria. Existing research on their detection includes different staining and molecular methods [33], but so far, none of them is in use in clinical practice. Cerca et al. [34] suggested the use of SYBR green (a component of the existing commercially available BacLight^TM^ LIVE/DEAD viability kit) as a fluorescent probe to assess the metabolic state of *S. epidermidis* bacteria by flow cytometry. Ou et al. [35] induced the VBNC state in MRSA strains with freezing at −20 °C, and subsequently evaluated their detection from contaminated food samples with the use of propidium monoazide-crossing priming amplification. Li et al. [36] successfully applied a propidium monoazide-polymerase chain reaction assay to rapidly detect VBNC *S. aureus* in food. A few studies also demonstrated that the detection of bacteria in the VBNC state with phages is possible. Awais et al. [37] engineered a GFP-labeled phage for the detection of *E. coli* and showed that also the detection of VBNC *E. coli* was possible with the method. Fernandez et al. [38] immobilized phage PVP-SE1 on the gold surface in a biosensor for the quantitative detection of VBNC *S. enteriditis*. They were able to successfully distinguish viable and VBNC cells from dead cells with a detection rate of 3–4 cells per sensor. As far as we know, no research on the detection of VBNC bacteria in biofilms formed by clinical isolates from patients with PJI using bacteriophages has been made.

*Staphylococcus aureus* and coagulase-negative staphylococci account for more than half of the cases of PJI, and the prevalence of methicillin-resistant *S. aureus* PJI is increasing [39,40]. Therefore, four clinically relevant and biofilm-forming staphylococcal strains—*S. epidermidis*, *S. lugdunensis,* and two *S. aureus* strains—were selected for this study. Importantly, two of the tested strains (*S. aureus* OBV 17/166 and *S. lugdunensis* OBV 20/143) were PJI clinical isolates. Bacteria were cultured for biofilm formation and transferred to low-nutrient media with three different gentamycin concentrations (4 × MIC, 8 × MIC, and 16 × MIC) for their induction into the VBNC state. We demonstrated that all tested strains were able to enter the VBNC state as early as 8 days and up to 19 days of exposure to gentamycin, regardless of the concentration used. To date, several foodborne and clinical bacterial species have been reported to be able to transition into the VBNC state, among them *S. aureus* and *S. epidermidis*. In the literature, different environmental conditions were used for induction. Pasquaroli et al. [18,19] successfully induced the VBNC state in *S. aureus* 10,850 biofilms with different concentrations of antibiotic vancomycin, quinupristin/dalfopristin, daptomycin, and/or nutrient depletion. Yan et al. [41] demonstrated the induction of the VBNC state in *S. aureus* in a citric acid buffer at −20 °C. Bacteria showed changes in biological characteristics and were able to resuscitate under many different conditions. Robben et al. [42] showed that non-ionic surfactants can induce the VBNC state in *S. aureus* in just about 5 min and up to 1 h of exposure. Cerca et al. [43] observed that pH and extracellular levels of calcium and magnesium induced the VBNC state in *S. epidermidis* biofilms. Most of the conducted research on VBNC bacteria is, however, still from the fields of the food industry and water monitoring. Furthermore, several human pathogens associated with medical device infection were also reported as being able to enter the VBNC state. Zandri et al. [44] analyzed 44 central venous catheters negative by the Maki technique (rolling the catheter segment across blood agar) for the presence of VBNC bacteria in the biofilm. An analysis of 39 culture-negative samples with fluorescent staining and a bacterial 16S rDNA analysis by qPCR confirmed the presence of VBNC bacteria in 77% of samples that did not grow on culture media, mostly from the *Staphylococci* spp. (*S. epidermidis* and *S. aureus*). However, the standard microbiology blood culture was positive only in 18% of patients. In a recent study, Wilkins et al. [45] demonstrated the presence of VBNC *P. aeruginosa*, *P. mirabilis,* and *E. coli* bacteria in biofilms forming on different antimicrobial urinary catheters, explaining why antimicrobial materials do not show a significantly important clinical improvement in vivo. However, we did not find any studies reporting the VBNC state in *S. lugdunensis*. Additionally, *S. epidermidis* has been reported in the literature as the only coagulase-negative *staphylococcus* to enter the VBNC state so far [46].

The results obtained in our study improved a previously developed method. The sensitivity of the method has already been determined previously by testing 104 clinical samples (sonicate fluid) with the method, and results were compared with the results obtained from microbiological conventional culturing. The sensitivity was 94.12%, with only one false negative result. However, in that patient, only conventional tissue culture was positive, while the culture of SF remained sterile [7]. For an exact determination of the method’s sensitivity in also detecting VBNC bacteria, a larger number of SF samples would need to be tested in an extended future research study, to sample patients with a confirmed PJI but negative microbiological results (cases suspicious for VBNC presence). The method could be additionally improved by the application of new bacteriophages, specific to other bacterial species, or even by the bacteriophages with a narrower host range, to distinguish between *S. aureus* and coagulase-negative staphylococci. Results could therefore contribute importantly to the development of a new fast and effective diagnostic tool for the detection of device-associated biofilm infections, and, more broadly, other difficult-to-diagnose infections.

## 4. Materials and Methods

### 4.1. Bacteria and Bacteriophages

*Staphylococcus aureus* subsp. *aureus* bacteriophage K ATCC 19685-B1™, *S. aureus* ATCC 25923, and *E. coli* ATCC 25922 were purchased from the American Type Culture Collection (ATCC, Manassas, Vancouver, BC, Canada). *E. coli* ATCC 25922 was used in the study only as a negative control in the detection with phage K and qPCR. *S. epidermidis* DSM 3269 was purchased from the German Collection of Microorganisms and Cell Cultures GmbH (DSMZ, Deutsche Sammlung von Mikroorganismen und Zellkulturen GmbH). Clinical isolates *S. lugdunensis* OBV 20/143 and *S. aureus* OBV 17/166 were from an »in-house«on-growing isolates library of Valdoltra Orthopaedic Hospital, and they were isolated from patients undergoing revision surgery at our institution due to PJI during routine microbiological diagnostic procedures. Species were determined by conventional microbiological culturing methods and confirmed by sequencing.

### 4.2. Biofilm Formation and Induction of VBNC State

For the preparation of M9 minimal nutrient agar, 200 mL of M9 salts solution, 20 mL of 20% glucose solution, 2 mL of 1 M MgSO_4_ (Carl Roth GmbH, Karlsruhe, Germany), 100 µL of 1 M CaCl_2_ (Fisher Scientific, Loughborough, UK), 14 g of Agar Bios Special LL (Biolife Italiana srl, Milan, Italy), and dH2O were combined for the preparation of 1 L media. The M9 salt solution was prepared by dissolving 56.8 g Na_2_HPO_4_ (Sigma-Aldrich, St. Louis, MO), 15 g KH_2_PO_4_ (Sigma-Aldrich, St. Louis, MO), 2.5 g NaCl (Carlo Erba, Milan, Italy), and 5 g NH_4_Cl (Honeywell Fluka, Seelze, Germany) into 1 L dH_2_O and sterilized by autoclaving. D-Glucose anhydrous (Fisher Scientific, Loughborough, UK) was used for the preparation of 1% and 20% glucose solutions, which were sterilized by filtration through 0.22 μm pore-size Minisart syringe filters (Sartorius Stedim Biotech GmbH, Goettingen, Germany).

For the selected bacteria, minimal inhibitory concentrations (MIC) for the antibiotic gentamycin were determined by the microdilution technique in 96-well microtiter plates according to the protocol described by Wiegand et al. [47]. Two-times dilutions of gentamycin (Sigma-Aldrich, St. Louis, MO) were prepared in BHI broth (Merck KGaA, Darmstadt, Germany) so that tested concentrations ranged from 100 µg/mL to 0.1 µg/mL. After the overnight incubation of plates at 37 °C, PrestoBlue™ Cell Viability Reagent (Invitrogen Life Technologies, Carlsbad, CA) was added to wells according to the manufacturer’s instructions, and fluorescence was measured with a Tecan Infinite 200 Pro MPlex plate reader (Tecan, Männedorf, Switzerland) to determine the proliferation of bacterial cells. Fluorescence values were plotted vs. antibiotic concentration, and MIC values were determined as the lowest concentrations of gentamycin, which prevented the visible growth of bacteria.

For biofilm development on a solid surface, the colony biofilm assay was performed [48,49]. A late logarithmic bacterial culture was diluted to match the 0.5 McFarland standard (Carl Roth GmbH, Karlsruhe, Germany), and a 100 µL of the diluted culture was spotted on a 0.22 μm sterile cellulose nitrate filter disc (Sartorius Stedim Biotech GmbH, Goettingen, Germany) placed on the BHI agar plate supplemented with 1% glucose. Plates were incubated at 37 °C for 48 h.

To induce the VBNC state in bacteria, each filter disc with biofilm was transferred to M9 minimal nutrient agar plates with gentamycin at 4 × MIC, 8 × MIC, and 16 × MIC. As a control, M9 minimal nutrient agar plates without gentamycin were used. Three parallel independent experiments were conducted for each tested bacterial strain under the same conditions. Plates were incubated aerobically at 37 °C and were weekly transferred to fresh plates of the same composition. The loss of culturability was monitored by sampling biofilms every other day and culturing biofilm samples on BHI agar plates at 37 °C for 7 days. The loss of culturability was determined if no visible growth was obtained after 7 days of culturing.

After the loss of culturability, biofilms were detached from filter paper discs by sonication. The applied sonication protocol is routinely used at our hospital for the sonication of explanted prosthetic devices for the microbiological assessment of PJI. Briefly, filter papers were placed in 5 mL sterile 0.85% NaCl solution, vortexed for 30 s, and then subjected to sonication at a frequency of 40 kHz and power density of 0.22 W/cm^2^ in a BactoSonicR ultrasonic bath (Bandelin GmbH, Berlin, Germany) filled with 4% Tickopur solution (TR3, Bandelin, Berlin, Germany) for 5 min, followed by additional vortexing for 30 s. The suspension was then transferred into a new 50 mL falcon tube (Isolab, Wertheim, Germany) and centrifuged for 15 min at 10,000× *g* using a Sigma 3–18 KS centrifuge (Sigma Laborzentrifugen GmbH, Osterode am Harz, Germany). Bacteria were resuspended in 5 mL of sterile 0.85% NaCl solution.

To determine the presence of VBNC bacteria in the detached biofilms, the LIVE/DEAD™ BacLight™ Bacterial Viability Kit (Invitrogen™, Molecular Probes Inc., OR, USA) was used according to the manufacturer’s instructions. Fluorescence was measured in black flat-bottom microtiter plates using a Tecan Infinite 200 Pro MPlex plate reader for the determination of viable cells percentages. Viable (green) and dead (red) bacteria were also visualized using a Fluorescence microscope Nikon Eclipse 80i (Nikon Corporation, Tokyo, Japan). Suspensions of live and dead bacterial cells were used as controls and were prepared as described in the BacLight™ kit manufacturer’s instructions. According to their protocol, 70% 2-propanol (Merck, Darmstadt, Germany) was used to kill bacteria by incubating at room temperature for 1 h and mixing every 15 min. Prepared suspensions were used also in subsequent qPCR experiments.

### 4.3. Detection of VBNC Bacteria with Bacteriophages and qPCR

Suspensions of VBNC, live, and dead bacterial cells were adjusted to OD_670nm_ of 0.6 diluting samples with the sterile 0.85% NaCl solution. A 100 µL of bacterial dilutions was then combined with BHI and bacteriophage K in a total volume of 1 mL. The final bacteriophage K concentration was 1.8 × 10^4^ PFU/mL. A reference control sample was assessed and consisted of BHI with phage K at the same concentration. As a negative control, live and dead *E. coli* ATCC 25922 suspensions were used. Samples were incubated at 37 °C with shaking for 180 min. After that, 30 µL of chloroform (Sigma-Aldrich, St. Louis, MO) was added to each sample and vortexed for 2 min. Afterward, samples were centrifuged in a tabletop MiniSpin centrifuge (Eppendorf AG, Hamburg, Germany) at 3287× *g* for 5 min, and 1 µL of each supernatant was assayed in the qPCR in triplicates.

During the qPCR, the phage’s DNA was quantified using previously designed [6] phage K-specific primers (forward 5’-CGTAGGTCACTCTCGTTTCG-3’ and reverse 5’-CGTCACCGTAGAATGAAGCC -3’) at a final concentration of 450 nM. DNA isolation prior to qPCR was previously proven unnecessary [6]. Reactions were performed in a total volume of 20 μL containing 10 µL of 2× Syber Green PowerUp master mix (Applied Biosystems, Life Technologies, Burlington, ON, Canada), 0.9 µL of each primer, 7.2 µL nuclease-free water, and 1 µL of the sample. The thermal cycling conditions were as follows: 2 min at 50 °C for UDG activation, 2 min at 95 °C for polymerase activation, and 45 cycles of 1 s at 95 °C and 30 s at 60 °C. In each qPCR run, a non-template control (using 1 μL nuclease-free water) as well as a reference control, containing only the initial amount of added phages, were included. The suspensions of live and dead bacterial cells with added phages were assessed as experimental controls. The protocols used for the detection of bacteria with phage K and qPCR, as well as the concentrations, were as optimized and defined in previous studies [6,7].

### 4.4. Statistical Analysis

The data shown are values obtained from three independent parallel experiments and expressed as mean ± SE (standard error) or mean ± SD (standard deviation). The SigmaPlot 12.0 (Systat Software, Chicago, IL, USA) program was used for the statistical analysis. A statistical analysis was performed by an analysis of variance (ANOVA, followed by the Student–Newman–Keuls test for comparisons across multiple groups) and Student’s t-test. Statistical significance was defined as *p* < 0.05.

## 5. Conclusions

We successfully inducted the VBNC state in the biofilms of *S. epidermidis*, *S. lugdunensis*, and two *S. aureus* strains with starvation and antibiotic pressure. With the tested method, we were able to specifically detect both live and VBNC staphylococci in a 4 h assay, through the detection of the increase or decrease in phage K DNA in the real-time PCR. Additionally, here, we first report the induction of the VBNC state in *S. lugdunensis*. To the best of our knowledge, *S. epidermidis* has been the only coagulase-negative *Staphylococcus* reported to enter the VBNC state up to today.

## Figures and Tables

**Table 1:** Results of VBNC state induction in four staphylococcal strains.

Bacterial Strain	Conditions for VBNC Induction	Days under Stress Conditions Until Loss of Culturability (Mean ± SE) ^1^	% of Viable Bacterial Cells after Culturability Loss (Mean ± SE) ^1^
*S. aureus* ATCC 25923	S	>30	-
	S + G 4 × MIC	11.3 ± 1.3	11.9 ± 1.5
	S + G 8 × MIC	8.0 ± 1.0	13.5 ± 0.4
	S + G 16 × MIC	9.0 ± 1.0	14.0 ± 0.7
*S. aureus* OBV 17/166	S	>30	-
	S + G 4 × MIC	19.3 ± 1.4	9.7 ± 0.1
	S + G 8 × MIC	19.3 ± 1.4	14.2 ± 0.0
	S + G 16 × MIC	13.7 ± 3.3	10.3 ± 0.9
*S. epidermidis* DSM 3269	S	>30	-
	S + G 4 × MIC	16.0 ± 0.0	6.1 ± 0.4
	S + G 8 × MIC	12.7 ± 1.3	10.7 ± 1.6
	S + G 16 × MIC	8.0 ± 1.0	12.0 ± 0.7
*S. lugdunensis* OBV 20/143	S	>30	-
	S + G 4 × MIC	15.3 ± 0.7	15.2 ± 3.5
	S + G 8 × MIC	14.0 ± 0.0	23.7 ± 0.5
	S + G 16 × MIC	10.0 ± 0.0	23.9 ± 0.5

S, starvation; G, gentamycin. ^1^ Values obtained from three independent parallel experiments and expressed as mean ± SE.

## Data Availability

The data presented in this study are available in the Results section of this article.
